# The Duration of Pregnancy

**Published:** 1914-05-30

**Authors:** 


### The Duration of Pregnancy.

The application of mathematics and mathematical methods to medical statistics and problems has become increasingly frequent in recent years, due mainly to the teachings of the eugenists. In the *Edinburgh Medical Journal* Dr. D. Berry Hart has applied these highly scientific methods to the old question of the duration of pregnancy and the calculation of the probable date of delivery. His results differ slightly from those hitherto accepted in text-books; possibly this may be due to the fact that only 500 cases have been taken, a number which may be too small to give absolutely reliable results in regard to so variable a phenomenon. The common practice is to reckon 280 days from the first day of the last menstrual period. Dr. Hart takes instead the last day of that period, and finds that the most probable duration of pregnancy reckoning from that date is 278¼ days. It will thus be seen that a calculation based on his plan would give a date some three, four, or five days later than on the other plan. We doubt the adoption of Dr. Hart's views, though he is evidently sure of his mathematics, for he has drawn up a revised obstetric table based on his calculations.

